# The Effectiveness of Mindfulness-Based Interventions on Maternal Perinatal Mental Health Outcomes: a Systematic Review

**DOI:** 10.1007/s12671-016-0673-y

**Published:** 2017-01-19

**Authors:** Zhenrong Shi, Angus MacBeth

**Affiliations:** Clinical and Health Psychology, School of Health in Social Science, Old Medical Quad, University of Edinburgh, Scotland, UK

**Keywords:** Mindfulness-based interventions, Mindfulness-based cognitive therapy, Mindful-based, Stress reduction, Mindfulness yoga, Perinatal, Anxiety, Depression, Pregnancy

## Abstract

Presenting with common mental health difficulties, particularly depression and anxiety, there is also preliminary evidence that mindfulness-based interventions (MBIs) including mindfulness-based cognitive therapy (MBCT), mindfulness-based stress reduction (MBSR) and integrated mindfulness yoga practices may also be effective in reducing common mental health difficulties during pregnancy. We systematically reviewed and synthesized the current literature on the effectiveness of MBIs in reducing severity of perinatal anxiety and depression. Databases including PubMed, Cochrane Library, IndMED and PsychoInfo were searched for relevant studies. Manual searches were conducted in relevant articles and Google Scholar. Seventeen cohorts representing 18 studies were included. Pre-post effect sizes were reported for both treatment and control groups. Seven randomized controlled trials (RCTs), two non-randomized controlled trials and nine treatment evaluations were included. Maternal participation in an MBI was associated with reductions in perinatal anxiety of moderate to large magnitude. Results for the effect of MBIs on depression were less consistent, with pre-post treatment reductions of moderate magnitude, but no significant differences in depression scores when MBI was compared with a control group. There was some evidence that MBIs were associated with increased mindfulness. Risk of bias in studies was variable. Our review offers preliminary evidence for the effectiveness of MBIs in reducing perinatal anxiety, with more equivocal findings with regard to perinatal depressive symptoms. Further methodologically rigorous evaluation using RCTs and longer follow-up periods are recommended.

## Introduction

Pregnancy and the postnatal period is a time of rapid and significant change in a women’s life, encompassing biological, social and psychological changes. Although often a time of positive emotion, estimates of the prevalence of anxiety and depression suggest at least 10% of pregnant women experience perinatal anxiety (Andersson et al. 2006), 20% of pregnant women suffer prenatal depression and between 12 and 16% of pregnant women are likely to suffer postnatal depression (Leung and Kaplan 2009). There is also substantial comorbidity between perinatal anxiety and depression (Di Florio et al. 2013; Grigoriadis et al. 2011). For instance, elevated anxiety during pregnancy is also considered as a risk factor for postpartum depression (e.g. Sutter-Dallay et al. 2004). Consequently, reducing perinatal psychological distress (anxiety, depression and stress occurring during pregnancy or in the first-year post-pregnancy) is a crucial public health goal.

There is increasing evidence that perinatal anxiety, depression and stress have both short- and long-term negative effects on mothers and newborns, with additional complex interactions between these variables (Staneva et al. 2015a). Both maternal anxiety, depression and stress increase risks for adverse neonatal outcomes including preterm birth (e.g. Dole et al. 2003; Glynn et al. 2008) and low birth weight (Dunkel Schetter and Lobel 2012; Hoffman and Hatch 2000) and small fetal head size (Field et al. 2010). These adverse outcomes are themselves linked to increased risk of infant mortality, neurodevelopmental impairment and disabilities (Wilson-Costello 2005) and adverse physical and mental health outcomes in later life (Sydsjö 2011). In addition, perinatal depression and anxiety may impact psychological development via changes to mother-infant interactions (e.g. Nicol-Harper et al. 2007), language development of children at 12 months (Quevedo et al. 2012) and behavioural, emotional and cognitive problems in middle childhood (Glover and O’Connor 2006; Huizink et al. 2003; O’Connor et al. 2002). From a treatment perspective, perinatal anxiety and depression may also be under-detected and untreated (Goodman and Tyer-Viola 2010). Furthermore, although antidepressant medication is effective in treating anxiety and depression, there is evidence of possible side effects of medication on neonatal outcomes including low birth weight, preterm birth, low Apgar scores, respiratory distress, neonatal convulsions and hypoglycemia (e.g. Grigoriadis et al. 2014; Hendrick et al. 2003; Huang et al. 2014; Kallen 2004). These risks indicate that development of effective non-pharmacological interventions in pregnancy would be beneficial.

A large number of studies suggest mindfulness-based interventions (MBIs) such as Mindfulness-based stress reduction (MBSR; Kabat-Zinn 2003) and Mindfulness-based cognitive therapy (MBCT; Segal et al. 2002) are effective psychological interventions to reduce depression and anxiety in clinical and non-clinical populations (Kuyken et al. 2015). MBIs have demonstrated effectiveness in preventing the recurrence of depression (Piet and Hougaard 2011; Segal et al. 2002), and MBSR has demonstrated effectiveness in reducing symptoms of both generalized anxiety (e.g. Hoge et al. 2013) and social anxiety (Koszycki et al. 2007). A recent meta-analytic review (Hoffman et al. 2010) reported a moderate effect size of MBIs on anxiety and mood reduction for all participants and a strong effect size for reducing anxiety (*g* = 0.97) and mood (*g* = 0.95) symptoms for those participants with pre-existing anxiety and mood disorders.

In addition, there is also an emergent evidence base for mindfulness-informed yoga interventions in pregnancy. With regard to general health in pregnancy, yoga integrated with a meditation intervention has been demonstrated to improve maternal physical health in pregnancy and improve labor and birth outcomes (Curtis et al. 2012; Narendran et al. 2005). There is also evidence that yoga practice in pregnancy reduces perinatal anxiety and depression (Newham et al. 2014). It is of note that non-pharmacologic interventions in pregnancy such as yoga and MBIs share overlapping common characteristics such as meditation and regulated breathing. Cramer et al. (2013) suggested that yoga and meditation may have effectiveness in the treatment of mental health difficulties. With specific reference to pregnancy, Gong et al. (2015) reviewed evidence that integrated yoga—including physical exercises, breathing (pranayama), meditation or deep relaxation—was effective in reducing prenatal depression. However, the results did not demonstrate the effectiveness of physical-exercise-based yoga. In addition, Beddoe et al. (2010) demonstrated that women in the third trimester reported significant anxiety and stress reductions after receiving mindfulness-informed yoga. Therefore, the evidence base for MBIs in perinatal mental health pregnancy could be enriched by considering yoga interventions that explicitly integrate mindfulness practice with yoga techniques (Muzik et al. 2012).

In summary, there is preliminary evidence that MBIs may be effective to anxiety and depression reduction for pregnant women (e.g. Vieten and Astin 2008, Woolhouse et al. 2014) and similar preliminary evidence regarding the effectiveness of yoga on reducing distress in pregnancy (Beddoe et al. 2010). There have been meta-analyses of mindfulness interventions in pregnancy (Hall et al. 2016; Taylor et al. 2016), focused on outcomes in common mental health symptoms, with both reviews highlighting issues with the quality of the data. However, these reviews varied in their approach to study designs, assessment of risk of bias and definitions of MBIs (including MBSR, MBCT and mindfulness-informed yoga). The literature on mindfulness in pregnancy also continues to accumulate at a rapid pace. We sought to systematically review the evidence for the effectiveness of MBIs (MBCT, MBSR and mindfulness-informed yoga) on common mental health difficulties (specifically anxiety, depression and stress) in pregnancy, with a focus on a narrative synthesis of the theoretical and methodological challenges in the current literature. Specifically, we hypothesized that MBIs would be effective in reducing levels of depression and anxiety both from pre-post treatment and compared to controls. We also hypothesized that there would be a broad range of methodological variance in the literature.

## Method

The review was conducted according to PRISMA guidelines (Moher et al. 2015. Four electronic bibliographic databases (PubMed, Cochrane Library, Ended and PsychInfo) were searched up to 28 September 2016. Database limits were set from 1980 to September 2016. Search terms were combined from conjunctions of the following terms: (‘mindfulness’ OR ‘mindfulness techniques’ OR ‘mindfulness approaches’ OR mindfulness-based cognitive therapy’ OR ‘mindfulness-based interventions/ or treatments’ OR ‘MBCT’ OR ‘mindfulness-based stress reduction’ OR ‘mindful yoga’ OR ‘mindful meditation’) AND (‘perinatal depression’ OR ‘peripartum depression’ OR ‘maternal depression’ OR ‘antenatal depression’ OR ‘prenatal depression’ OR ‘pre-partum depression’ OR ‘post-partum depression’ OR ‘postnatal depression’) OR (‘perinatal anxiety’ OR ‘peripartum anxiety’ OR ‘maternal anxiety’, OR ‘antenatal anxiety’). Manual searches were conducted for cross-references in relevant articles and review papers extracted from the database searches and in Google Scholar by using the combination of the above terms. An expert librarian was consulted with regard to the search terms.

Our inclusion criteria were for female participants meeting the following criteria: participants were either primigravida or multigravida; measurement of depression and/or anxiety symptoms was implemented using either validated self-report or interview measures; or participants met diagnostic criteria for a depressive or anxiety disorder based on criteria from the Diagnostic and Statistical Manual, Fourth Edition (DSM-IV) (American Psychiatric Association 2000) or the International Classification of Disease 10 (ICD-10) criteria (World Health Organization, 1993). Participants were assessed either during pregnancy or during first year after delivery, aged between 16 and 45 years old and could speak and read English. In addition, studies were selected if they compared MBI with a control group (either treatment without therapist, treatment as usual or care as usual or waiting-list control conditions) or without a control group. Study designs were either randomized controlled trials (RCTs), non-randomized controlled trials, or non-controlled trials with quantitative data. Finally, studies were included if their treatment component used either manualized protocols or accredited facilitators or was delivered by health professional with specific training in facilitation of MBIs.

Studies were excluded if participants had current psychosis or other complex mental disorders, where depressive and/or anxiety symptoms were comorbid symptoms of a specific physical disorder and where women were a priori identified as medically defined high-risk pregnancies (e.g. multiple pregnancies). In addition, we excluded qualitative studies, case studies, book chapters and literature reviews. No restrictions were made in terms of the participants’ attendance rate of the mindfulness-based interventions, ethnic origin, marital status, weeks of gestation and previous experience of MBIs.

Interventions were eligible for inclusion if they included an MBI such as MBSR or MBCT. We included yoga interventions only where there was clear evidence from the intervention description that the intervention included several components consistent with integrated mindfulness practice (e.g. techniques to encourage a non-judgemental focus on sensation experienced in the current moment, meditation, breathing, body scan, deep relaxation), rather than simply a description of yoga practices per se. We therefore included studies with an explicit statement that the yoga intervention included integrated mindfulness practice. Interventions without detailed description of its components were excluded. No restrictions were made regarding the length, frequency or duration of the MBI. Included studies had to examine at least one of the primary outcomes: anxiety and depression. Secondary outcomes of interest were stress and mindfulness. Inclusion of studies was initially made by the first author. Where there was uncertainty regarding inclusion, queries were resolved by consensus discussion with the second author.

An adaption of the SIGN 50 Methodology Checklist (Scottish Intercollegiate Guideline Network checklist 2015) was used to extract study characteristics including specific details about the study design, population, interventions, follow-up, outcome measures and results. For studies which providing the mean scores and standard deviations of baseline as well as post-interventions, effect size (ES) Cohen’s *d* and their 95% confidence intervals were calculated. For controlled studies, ES was calculated for the differences between pre- and post-interventions in both the treatment and control groups. For non-controlled studies, ES was also calculated in treatment group to compare the changes from baseline to post-interventions. Effect sizes (ESs; Cohen 1988) were divided into five levels: trivial (*d* ≤ 0.2), small (*d* > 0.2), moderate (*d* > 0.5), large (*d* > 0.8), and very large (*d* > 1.3). The revised Cochrane risk of bias tool was used to evaluate risk of bias of included studies (Higgins et al. 2011). The studies were rated according to five domains: selection bias, performance bias, detection bias, attrition bias and reporting bias. The first author assessed the risk of bias for all studies. Inter-rater reliability was calculated by the second rating of a randomly selected 40% of studies by an independent investigator, blind to review aims. The inter-rater reliability was 0.80 (Cohen’s kappa), indicating high agreement between the two reviewers on risk of bias assessments.

## Results

**Fig. 1:**
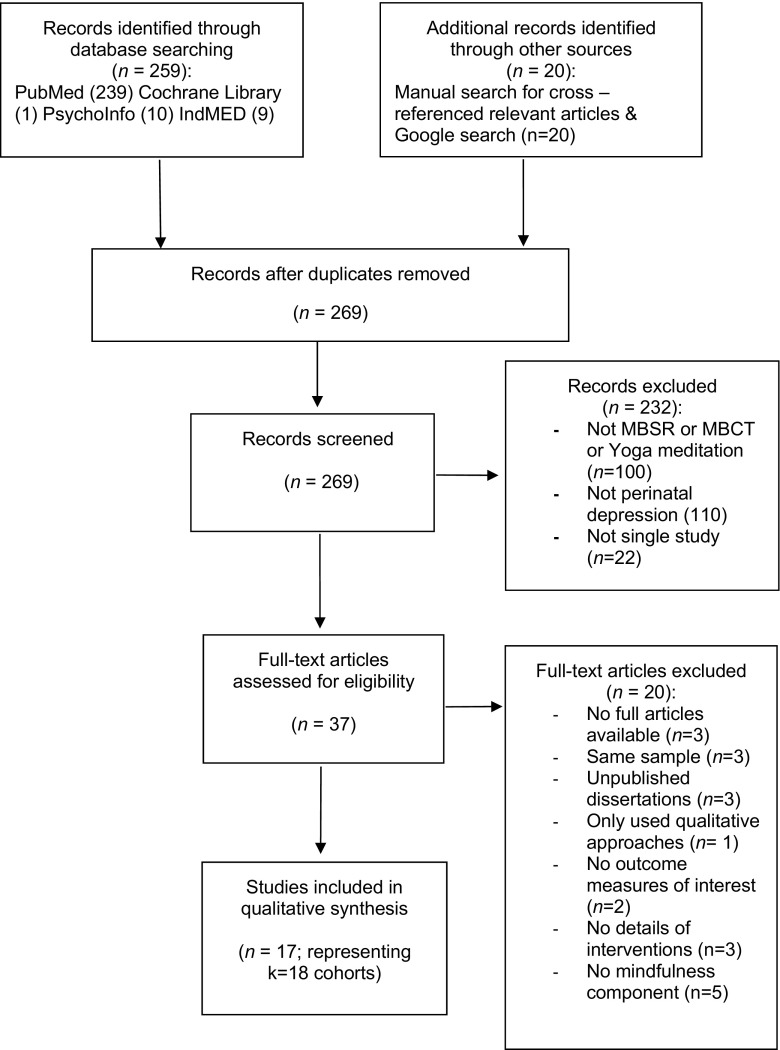
PRISMA flow chart for identification and selection of studies

Procedures for screening of studies are displayed in Fig. 1. The final data set consisted of 17 studies reporting results from *k* = 18 cohorts (Fig. 1). One study (Woolhouse et al. 2014) reported results from two distinct samples (hereafter labelled samples 1 and 2). A summary of study characteristics of the 18 included cohorts is presented in Table 1. Of the included studies, seven studies were randomized controlled trials (Dimidjian et al. 2016; Guardino et al. 2014; Narimani and Musavi 2015; Perez-Blasco et al. 2013; Vieten and Astin 2008; Woolhouse et al. 2014, sample 1; Zhang and Emory, 2015); two studies were non-randomized controlled trials (Dunn et al. 2012; Miklowitz et al. 2015) and nine studies were non-controlled trials (Battle et al. 2015; Beddoe et al. 2010; Byrne et al. 2014; Dimidjian et al. 2015; Duncan and Bardacke 2010; Felder et al. 2016; Goodman et al. 2014; Muzik et al. 2012; Woolhouse et al. 2014, sample 2). Of the included five RCTs, the control group types were waiting-list control (*n* = 2; Perez-Blasco et al. 2013; Vieten and Astin 2008); care-as-usual (*n* = 4; Dimidjian et al. 2016; Dunn et al. 2012; Woolhouse et al. 2014, sample 1; Zhang and Emory, 2015); no intervention (*n* = 1; Narimani and Musavi 2015) and reading control (*n* = 1; Guardino et al. 2014).

**Table. 1:** Summary of included studies’ characteristics

Study (author, year, country)	Design	Participants Total sample size (*N*) Analyzed sample size (*n*) Mean age (SD) Mean gestation (SD or range)	Ethnicity	Education	Marital status	Type of sample (mental health, general population) setting	Treatment group (*n*)	Control group (*n*)	Duration of treatment	Outcome domains: Depression Anxiety Mindfulness	Outcome measures	Dropout rates (as fraction of total sample)	Length of follow-up	Attendance mean (s.d.)
Vieten and Astin (2008), USA	RCT	*N* = 34 *n* = 31 *M* = 33.90 (3.80) *M* = 25 weeks of gestation (between 12th and 30th)	Asian: *n* = 2 Hispanic: *n* = 4 Caucasian: *n* = 23 Other: *n* = 2	Mean educational level was 17 years	All married	Mental health Past history of treated psychiatric disorder (35%) Psychotropic medications (32%) Score >16 on CES-D (31%) Score >14 on PSS (52%) Urban Hospital	MBI (mindful motherhood)	W/L (*n* = 18)	8× weekly 2-h sessions	Depression Anxiety Mindfulness	CES-D STAI MAAS	Intervention group (2/15) Control group (1/19)	3 months	7.2 sessions (1.1)
Beddoe et al. (2010), USA	TxE	*N* = 23 *n* = 16 *M* = 30.4 Between 12th and 32nd weeks gestation	N/R	N/R	All married	General population with screening for history of depression and anxiety (*N* = 5, 1/3). No Current medical problems N/R	MBI (MBSR + Yoga)	N/A	7× weekly intervention	Trait anxiety State anxiety	STAI-T STAI-S	7/23	No	N/R
Duncan and Bardacke (2010), USA	TxE	*N* = 35 *n* = 27 *M* = 34.6 (4.22) Between late 2nd trimester and early 3rd trimester	Asian/Pacific Islander: *n* = 1 Hispanic: *n* = 1 Caucasian: *n* = 24 Other: *n* = 1	College: *n* = 2 Bachelor’s degree: *n* = 8 Graduate school: *n* = 3 Masters/doctoral degree: *n* = 14	All couples	General population. University clinic/off site location	MBI (mindfulness-based childbirth and parenting education)	N/A	10 weeks (class sessions 1–9 and class reunion)	Depression Pregnancy anxiety Mindfulness	CES-D, revised-PAS, FFMQ	8/35	Qualitative follow-up, but time not reported	8.3//10
Dunn et al. (2012) Australia	NCT	*N* = 19 Treatment group: *M* = 35.33 (4.53) Control group: *M* = 27.67 (5.43) Between 12th and 29th weeks of gestation	White: *n* = 61 Black: *n* = 16 Asian: *n* = 2 Hispanic: *n* = 6 Other: *n* = 1	N/R	9/10 in committed Rx	General population on past history of anxiety and/or depression in intervention group (9/10) Control group (0/9) Women’s and Children’s Hospital	MBCT modified for pregnancy (*n* = 10)	CAU (*n* = 9)	8× weekly 2-h sessions	Depression Anxiety Mindfulness	EPDS DASS21 MAAS	No details	6-weeks post-partum.	N/R
Muzik et al. (2012), USA	TxE	*N* = 22 *n* = 18 *M* = 32.41 (4.98) *M* = 21.80 weeks (5.96) ≤26 weeks gestation	Caucasian: *n* = 15 Other: *n* = 6	<Bachelor’s degree: *n* = 3 Bachelor’s degree: *n* = 7 Masters/doctoral degree: *n* = 12	5/22 single; 17/22 living with partner	General population with mental health screening: No diagnosis 50.0% (*n* = 11), Major depression 9.1% (*n* = 2), Post-traumatic stress disorder 18.2% (*n* = 4); Anxiety disorder (GAD, phobias, panic disorder) 45.5% (*n* = 10) N/R	MBI (mindful yoga)	N/A	10× weekly 1.5-h sessions	Depression Mindfulness	EPDS BDI-II SCID I FFMQ-revised	4/22	No	7.83 (1.62)
Perez-Blasco et al. (2013)	RCT	*N* = 22 *N* = 21 *M* = 34.33 (4.72) Postnatal (mean age of infants = 10.75 months)	N/R	N/R	N/R	General population. Community Health Center	MBI (composite program of MBSR, MBCT and mindful self-compassion)	W/L control	8× weekly 2-h sessions	Depression Anxiety Stress	DASS-21D DASS-21A DASS-21S	0/13 Tx group; *N* = 5/13 participants from control group	No	N/R
Byrne et al.9 (2014), Australia	TxE	*N* = 18 *n* = 12 *M* = 30.1 (3.7) Between 18th and 28th weeks	N/R	Undergraduate degree: *n* = 10 Masters/doctoral degree: *n* = 4	2/18 single; 16/18 living with partner	General population	MBI (mindfulness-based childbirth education program) N/R	N/A	8× weekly 2.5-h sessions	Depression Anxiety Mindfulness	EPDS DASS-21 MAAS	6/18	Approximately 3 to 12 weeks postpartum	N/R
Goodman et al. (2014), USA	TxE	*N* = 24 *n* = 23 *M* = 33.5 (4.40) range 27–45 years old *M* = 15.54 weeks of gestation (5.83) Between 6th and 27th weeks	Asian/Pacific Islander: *n* = 3 Hispanic: *n* = 2 Caucasian: *n* = 18 Other: *n* = 1	College: *n* = 3 College degree: *n* = 6 Graduate degree or above: *n* = 15	1/24 single; 23/24 living with partner	Mental health Met criteria for GAD (*n* = 17, 70.8%) Fail to meet criteria, but with elevated level of generalized anxiety and worry symptoms (*n* = 7, 29.2% ). Academic institution	MBCT (CLAM pregnancy intervention)	N/A	8× weekly 2-h sessions	Depression Anxiety Mindfulness	BDI-II MINI BAI MAAS	1/24	No	6.96
Guardino et al. (2014), USA	RCT	*N* = 47 *M* = 33.13 (4.79) *M* = 17.78 weeks (5.10) Between 10th and 25th weeks	Caucasian = 31	4 year university degree or greater: *n* = 42	44/47 living with partner	General population But screened for mental health issues. S/R previous diagnosis of depression (30%) of anxiety disorder (31%) of other psychological disorder (10%). Academic center	MBI (mindful awareness practices program)	Reading control: ‘You and Your Baby: Pregnancy’ book, Riley, 2006)	6× weekly 2-h sessions	Anxiety Pregnancy-specific anxiety Mindfulness	STAI PSA PRA FFMQ	Intervention group (4/24) Control group (10/23)	6 weeks	4.75 (1.07)
Woolhouse et al. (2014), sample 1, Australia	RCT	*N* = 32 *n* = 23 *M* = 32.39 (0.65) Treatment group: *M* = 30.81 (0.75) Control group: *M* = 34.08 (0.90) Between 1st and 3rd trimesters	N/R	Below university education: *n* = 5 Undergraduate degree: *n* = 13 Masters/doctoral degree: *n* = 14	1/32 single; 21/32 married; 10/32 living with partner	General population Maternity hospital	MBI (mind baby body intervention) (*n* = 17)	CAU (*n* = 15)	6× weekly 2-h sessions	Depression Anxiety Mindfulness	CES-D DASS21 STAI FFMQ	Intervention group (4/17) Control group (5/15)	No details	N/R
Woolhouse et al., (2014), sample 2, Australia	TxE	*N* = 20 *M* = 33.70 (1.29) Between 2nd and 3rd trimester	N/R	Below university education: *n* = 5 Undergraduate degree: *n* = 10 Masters/doctoral degree: *n* = 5	2/20 single; 16/20 married; 2/20 living with partner	Mental health Currently experiencing or identified at risk of stress, anxiety and depression. Maternity hospital	MBI (mind baby body intervention	N/A	6× weekly 2-h sessions	Depression Anxiety Mindfulness	CES-D STAI DASS21 FFMQ	9/20	No details	N/R
Battle et al. (2015), USA	TxE	*N* = 34 *n* = 22 *M* = 28 (5.4) Range 19–40 years old. *M* = 19.0 weeks gestation (4.7) Between 12th and 26.5th weeks gestation	Caucasian: *n* = 14 Black/African American: *n* = 5 Multiracial: *n* = 2 Other: *n* = 11	High School not completed: *n* = 3 High School: *n* = 5 College: *n* = 9 Bachelor’s Degree: *n* = 17	23/34 married/living with partner 11/3 single/divorced	Mental health Current MDD 25 (74%) MDD earlier in pregnancy 4 (12%) Current minor depressive disorder 4 (12%) Minor depressive disorder earlier in pregnancy 1 (3%). N/R	MBI (prenatal yoga)	N/A	10× weeks Unknown each session’s length	Depression Mindfulness	SCID-IP EPDS QIDS FFMQ	12/34	No	5.2 (3.7)
Dimidjian et al. (2015), USA	TxE	*N* = 49 *M* = 31.83 (4.19) *M* = 17.25 weeks gestation (6.90)	Asian/Pacific Islander: *n* = 2 African American: *n* = 3 Hispanic: *n* = 2 Caucasian: *n* = 40 Other: *n* = 2	N/R	46/49 married	Mental Health A history of depression (100%) One episode (47%) Two episodes (29%) Three or more episodes (24%) Lifetime alcohol/substance abuse or dependence (33%) Current or lifetime anxiety disorder (31%). Health Clinic	Modified MBCT	N/A	8× weekly 2-h sessions (with monthly follow-up class)	Depression	EPDS	7/49	1st and 6th month	6.10 (.199)
Narimani and Musavi (2015), Iran	RCT	*N* = 30 Under 20 years, range 16–19 years. No details of gestation	N/R	N/R	N/R	Mental health “High scores on DASS21” N/R	MBCT	No intervention (*n* = 15)	8× weekly 2-h sessions	Depression Anxiety	DASS21 STAI	No details	No	N/R
Miklowitz et al. (2015)	NCT	*n* = 39 (*n* = 27 with depression) *n* = 39 35.2 (5.2) Perinatal status: Pre-conception = 7 Pregnant = 6 Postpartum = 14	Asian/Pacific Islander: *n* = 1 African American: *n* = 1 Hispanic: *n* = 1 Caucasian: *n* = 21 Other: *n* = 1	College: *n* = 2 2 year college degree: *n* = 1 4-year college degree: *n* = 10 Post-baccalaureate: *n* = 14	N/R	Mental health DSM-IV diagnosis of MDD and current subthreshold symptoms of depression. University center	MBCT	Comparison to group with diagnosis of bipolar disorder (*n* = 12)	8× weekly 2-h sessions	Depression	BDI-II HRSD FFMQ	8/39 at 6 months assessment Intention to treat analysis	6 month follow-up	82.1% completed more than 50% of sessions
Zhang and Emory (2015)	RCT	*N* = 65 *N* = 33 *M* = 25.3 (4.6) 12–31 weeks gestation	African American: *N* = 65	N/R	19/65 single 25/65 living with partner 12/65 not living with partner 9/65 married	General population, targeted to areas of low income; ethnic minorities N/R	MBI (mindful motherhood)	TAU (*n* = 31)	8× sessions over 4 weeks	Depression Stress Mindfulness	EPDS PSS TMS	33/65 17/34 from Tx 14/31 from TAU	1 month post Tx	6/33 completed more than 7 sesssions
Dimidjian et al. (2016)	RCT	*n* = 86 *n* = 86 MBCT-PD: *M* = 30.98 (4.08) TAU: *M* = 28.72 (5.50) Perinatal status: Up to 32 weeks gestation	White: *n* = 61 Black: *n* = 16 Asian: *n* = 2 Hispanic: *n* = 6 Other: *n* = 1	College graduate: MBCT-PD group: *N* = 36 TAU: *n* = 30	MBCT: 38/43 Married/cohabiting TAU: 35/43 married/cohabiting	Previous prior MDD, but not in last 2 months. Health clinic	MBCT-PD	TAU (*n* = 43)	8-session manualised protocol Yoga DVD	Depression	EPDS LIFE (depressive relapse)	17/86 11/43 in MBCT group 6/43 from TAU Intention to treat analysis	6 months postpartum	6.89 (2.04)
Felder et al. (2016)	TxE	*n* = 37 *n* = 37 30.49 (4.09) 24.53 weeks (7.81)	White: *n* = 28 Black: *n* = 2 Asian: *n* = 1 Hispanic: *n* = 4 Other: *n* = 2	College graduate = 29	34/37 married/cohabiting	Previous prior MDD, but not in last 2 months. Health clinic (web delivered)	MBCT (MMB)	N/A	8-session manualised web-based protocol Yoga DVD	Depression	EPDS	16/37 Intention to treat analysis Lost to follow-up *n* = 12 Discontinued intervention: *n* = 4	Unclear	4.72

*Notes*: Individuals who were experiencing or at risk of stress, anxiety and depression were identified as mental health samples. In contrast, individuals without current depression or anxiety were identified as general population; *Depression* depressive symptoms, *RCT* randomized controlled trials, *NCT* non-randomized controlled trial, *TxE* within-subject treatment evaluation, *MBI* mindfulness-based interventions, *MBSR* mindfulness-based stress reduction, *MBCT* mindfulness-based cognitive therapy, *W/L* waiting list control, *CAU* care as usual, *S/R* self-reported, *mins* minutes, *2-h* 2 h, *DASS21* Depression, Anxiety, and Stress Scale (Lovibond and Lovibond 1995), *MAAS* Mindful Attention and Awareness Scale (Brown & Ryan, 2003), *PSA* pregnancy-specific anxiety (Roesch et al. 2004), *PRA* (Rini et al. 1999); *FFMQ* The Five Facet Mindfulness Questionnaire (Baer et al. 2006), *BAI* The Beck Anxiety Inventory (Beck and Steer 1990), *BDI-II* The Beck Depression Inventory—second edition (Beck et al. 1996), *EPDS* Edinburgh Post Natal Depression Scale (Cox et al. 1987), *STAI-T* The trait subscale of the State-Trait Anxiety Inventory (Spielberger 1989), *STAI-S* The state subscale of the State-Trait Anxiety Inventory (Marteau and Bekker 1992), *PAS* Pregnancy Anxiety Scale (Levin 1991); *PSS* Perceived Stress Scale (Cohen et al. 1983), *CES-D* Center for Epidemiological Studies Depression Scale (Radloff 1977; Hann et al. 1999), *QIDS* Quick Inventory of Depressive Symptomatology (Rush et al. 2006), *MDD* major depressive disorder, *LIFE* Longitudinal Interval Follow-up Evaluation (Keller et al. 1987), *N/R* not reported, *MMB* mindful mood balance, *DASS-21D* Depression, Anxiety and Stress Scale-Depression Subscale, *DASS-21A* Depression, Anxiety and Stress Scale-Anxiety Subscale, *DASS-21S* Depression, Anxiety and Stress Scale-Stress Subscale, *TMS* Toronto Mindfulness Scale, *Rx r*elationship

In terms of study settings and participant characteristics, there were *n* = 640 participants enrolled in the included studies. After excluding participants who dropped out of the intervention programs or failed to finish post-intervention assessments, findings from *n* = 603 participants were reported. Twelve studies were conducted in the USA (Battle et al. 2015; Beddoe et al. 2010; Dimidjian et al. 2015, 2016; Duncan and Bardacke 2010; Felder et al. 2016; Goodman et al. 2014; Guardino et al. 2014; Miklowitz et al. 2015; Muzik et al. 2012; Vieten and Astin 2008; Zhang and Emory 2015); four studies in Australia (Byrne et al. 2014; Dunn et al. 2012; Woolhouse et al. 2014, both samples); one in Iran (Narimani and Musavi 2015) and one in Spain (Perez-Blasco et al. 2013). Of the included studies, sixteen studies involved adult pregnant women, one study involving pregnant adolescents who were less than 20 years old and one recruited women in the first-year post-pregnancy. All included studies reported the mean age; most studies involved adults with mean maternal age ranged from 30 to 35 years old (*n* = 15). Fifteen studies reported the mean gestation at the start of the intervention, while three studies did not report the details of gestation. Across studies, the mean gestation ranged from the first trimester to middle 3rd trimester. Nine studies involved mental health samples of participants who were experiencing or identified at risk of stress, anxiety and/or depression. Eight studies involved participants who were non-depressed and anxious, healthy women recruited from the general population, although three of these studies involved screening for mental health difficulties. One study targeted women from low-income, ethnic minority areas (Zhang and Emory 2015). With the exception of two studies (Narimani and Musavi 2015; Zhang and Emory 2015), all studies reported the majority of participating women to be married, cohabiting or living together. Studies were conducted in a variety of settings from university clinics, maternity hospitals and general clinics.

With regard to treatment identified MBIs included variants on MBCT, MBSR and mindfulness yoga. Seven studies used MBCT (Dimidjian et al. 2015, 2016; Dunn et al. 2012; Felder et al. 2016; Goodman et al. 2014; Miklowitz et al. 2015; Narimani and Musavi 2015). Nine studies used MBSR or variants (Guardino et al. 2014; Vieten and Astin 2008; Woolhouse et al. 2014, both samples; Beddoe et al. 2010; Byrne et al. 2014; Duncan and Bardacke 2010; Perez-Blasco et al. 2013; Zhang and Emory 2015). One study involved mindfulness yoga (Muzik et al. 2012), and the other one used prenatal yoga (Battle et al. 2015). Mean duration of treatment was 8 weeks (range = 6 to 10 weeks). The mean session length was 2 h (range = 1.5 to 2.5 h). All sessions were led by trained instructors, clinical psychologists or certificated therapists. Engagement with treatment in most studies was high, particularly for MBCT- and MBSR-based approaches. One study (Zhang and Emory 2015) reported low levels of engagement throughout the treatment program.

The outcome measures used for assessment of depression and anxiety varied between studies. All included studies used self-report measures to assess depression and anxiety symptoms. The 18 included studies conducted baseline assessment and immediate post-treatment assessment. In addition, eight studies conducted post-treatment follow-up assessments. The timing of assessment ranged from 3 weeks to 6 months postpartum.

The effectiveness of MBIs upon depressive symptoms was examined in 16 studies (Table 2). With regard to controlled studies, of the six RCTs, three showed significant post-treatment reductions in depressive symptoms for MBCT compared to controls (Dimidjian et al. 2016; Narimani and Musavi 2015; Zhang and Emory 2015). Two RCTs showed trends toward post-treatment improvement for MBIs, based on self-report measures of depression (Vieten and Astin 2008; Woolhouse et al. 2014, sample 1). One study showed no difference between groups (Perez-Blasco et al. 2013). In the non-randomized trials, one study (Dunn et al. 2012) reported clinically significant different scores with inconclusive results for reduction in depression, while one study reported reductions in depressive symptoms for MBCT (Miklowitz et al. 2015). For within-group changes, the four RCTs, two reported large ESs (*d* = 0.70; Dimidjian et al. 2016; *d* = 0.83; Perez-Blasco et al. 2013) and three reported small to moderate ESs (*d* = 0.53; Vieten and Astin 2008; *d* = 0.30 and *d* = 0.54 for CES-D and DASS-32, respectively; Woolhouse et al. 2014, sample 1; Zhang and Emory 2015).

**Table. 2:** Key outcomes on depression, anxiety and stress for included studies

Cohort	Treatment group					Control group				Key findings
	*N* of sample: baseline/post intervention	Outcomes: Depression Anxiety Stress	Baseline mean (S.D.)	Post intervention mean (S.D*.*)	ES 95% CI	*N* of sample: baseline/post intervention	Baseline mean (S.D.)	Post intervention mean (S.D.)	ES 95% CI	
Results for depressive symptoms										
RCTs										
Vieten and Astin (2008)	13/13	CES-D STAI-S	20.40 (8.40) 43.8 (12.4)	16.20 (7.30) 35.4 (9.1)	0.53 [−1.32–0.25] 0.77 [−1.57–0.03]	18/18	14.20 (5.40) 35.6 (10.9)	17.20 (7.40) 35.6 (8.4)	0.46 [−0.20–1.13] 0 [−0.65–0.65]	No significant improvement found in Tx, compared to the CG (*p* = .06), but trend toward significant intervention
Perez-Blasco et al. (2013)	13/13	DASS-21D	4.46 (2.60)	2.31 (2.56)	0.83 (0.80–0.86)	13/8	7.00 (9.50)	3.50 (3.96)	0.55 (0.01–0.89)	Large within-subjects reduction in depression scores for Tx, but no difference between groups
Woolhouse et al. (2014), sample 1	13/13	CES-D	14.42 (10.05)	12.08 (4.17)	0.30 [−1.08–0.47]	10/10	13.70 (8.00)	10.10 (8.72)	0.43 [−1.32–0.46]	For TX, both post-intervention CES-D scale and DASS21 depression subscale scores improved, but not achieving statistical significance.
		DASS21	7.23 (6.66)	4.31 (3.64)	0.54 [−1.33–0.24]		8.00 (11.20)	5.60 (8.32)	0.24 [−1.12–0.64]	
Narimani and Musavi (2015)	15/15	DASS21*	N/R	N/R	N/C N/C	15/15	N/R	N/R	N/C N/C	MBCT was significantly effective upon depression and anxiety in pregnancy for women aged below 20 years (*p* < .0005)
Zhang and Emory (2015)	34/16	BDI-II	18.9 (11.2)	17.3 (10.2)	0.15 (−0.29–0.59)	30/17	14.2 (8.97)	15.2 (7.70)	−0.11 (−0.82–0.59)	No difference between Tx and TAU at post-intervention; decrease in depressive symptoms for Tx group at 1 month follow-up
Dimidjian et al. (2016)	*43/43*	EPDS	5.98 (3.95)	4.67 (3.95)	0.70	*43/43*	5.07 (4.91)	6.39 (3.81)	−0.54	MBCT associated with reduced symptoms compared to TAUY, held to followup. MBCT associated with significant reduction in relapse rates
Non-randomized controlled trials										
Dunn et al. (2012)	4/4	DASS 21	N/R	N/R	N/C N/C	5/5	N/R	N/R	N/C N/C	1 participant out of 4 participants showed clinical reliable improvement of depression (EPDS) in TX, No participant showed reduction of depression (EPDS) in CG. Reversed results were found on DASS-depression scale (no participant to improve depression in TX vs. 1 participant to improve depression in CG)
Miklowitz et al. (2015)	27/25	BDI-II HRSD	14.2 (10.3) 6.1 (4.8)	N/R N/R	0.74 0.36	*12/7*	7.7 (8.9) 3.8 (3.6)	N/R N/R	−.27 −.73	MBCT led to reductions in depression scores at follow-up, with a recurrence rate of 21.9% for depression
Treatment evaluations										
Duncan et al. (2010)	27/27	CES-D	1.63 (0.45)	1.48 (0.34)	0.38 [−0.91–0.16]					Significant reductions on the CES-D depression scale (*p* = .016).
Muzik et al. (2012)	18/18	BDI-II	13.95 (6.84)	9.63 (6.99)	0.63 [−1.29–0.04]					Significant reductions on both the BDI-II (*p* = .025) and EPDS depression scale (*p* = .001)
		EPDS	12.45 (3.41)	7.60 (4.16)	1.23 [−1.99–0.56]					
Goodman et al. (2014)	23/23	BDI-II	11.87 (5.67)	6.39 (6.36)	0.91 [−1.52–0.30]					Significant improvements on the BDI-II depression scale (*p* < .001).
Byrne et al. (2014)	12	EPDS	7.33 (5.07)	7.00 (2.83)	0.08 [−0.88–0.72]					No significant improvements on EPDS depression scale (*p* = .42). The depression (DASS-21) trended toward improvement, but not reaching statistically significant (*p* = .07)
		DASS21	5.83 (5.29)	3.17 (3.46)	0.60 [−1.41–0.22]					
Woolhouse et al. (2014), sample 2	11/11	CES-D	24.60 8.19	18.20 9.13	0.74 [−1.60–0.13]					Significant improvements on CES-D (*p* = 0.04), and DASS21 depression scale (*p* = 0.01)
		DASS21	13.80 7.74	9.60 6.10	0.60 [−1.46–0.25]					
Battle et al. (2015)	22/22	QIDS	12.6 (3.2)	N/R	N/C N/C					Significant decreases in depression symptoms over time on both the QIDS and the EPDS. Over 10 weeks, women’s symptoms decreased, on average, 4.4 points on the QIDS (S.D*.* = 1.40) and 5.5 points on the EPDS (S.D*.* = 1.00). Authors note clinically significant reductions in symptoms
		EPDS	13.0 (5.3)	N/R	N/C N/C					
Dimidjian et al. (2015)	49/49	EPDS	N/R	N/R	0.71 N/R					Significant reductions in depression symptoms observed during the intervention was sustained throughout the perinatal period on the EPDS scores, relative to baseline during pregnancy and postpartum (*p* = 0.013). Relapse rate of 18.37% in the sample from pregnancy to 6-month follow-up
Felder et al. (2016)	37/21	EPDS PHQ-9	N/R	N/R	N/C					No significant reduction in depression scores using PHQ-9 (*p* = .76) or EPDS (*p* = .67)
Results for anxiety symptoms										
RCTs										
Vieten andAstin. (2008)	13/13	STAI-S	43.8 (12.4)	35.4 (9.1)	0.77 [−1.57–0.03]	18/18	35.6 (10.9)	35.6 (8.4)	0 [−0.65–0.65]	Participants in TG showed statistically significant reduction in state anxiety compared with wait-list CG (*p* = .04). The CG showed no improvement in anxiety at the post-intervention assessment.
Perez-Blasco et al. (2013)	13/13	DASS-21A	7.08 (7.19)	2.46 (3.38)	0.82 (0.80–0.84)	13/8	7.50 (8.12)	7.25 (4.40)	0.03 (−0.84–0.88)	Large within-subjects reduction in anxiety scores for Tx; significant decrease in anxiety in Tx group compared to controls
Guardino et al. (2014)	24/21	PSA	11.63 (2.96)	7.65 (1.73)	1.60 [−2.29 - -0.94]	23/20	10.7 (2.79)	8.95 (3.0)	0.61 [−1.22–0.01]	A significantly larger decrease in PSA scores in TG (*p* < .05) than in CG (*p* < .05) over time. A significant reduction in PRA scores in TG (*p*<. 05), but not in the CG (*p* > .05) between pre- and post-intervention. State anxiety also decreased in the TG, but the changes were not significantly different from changes in CG
		PRA	24.42 (3.79)	22.7 (3.84)	0.45 [−1.04–0.14]		23.22 (4.95)	22.65 (5.93)	0.11 [−0.71–0.50]	
		STAI	45.69 7.64	39.47 6.27	0.88 [−1.50 - -0.27]		44.37 10.98	37.35 11.51	0.63 [−1.24–0.01]	
Woolhouse et al. (2014), sample 1	13/13	STAI	35.92 (14.11)	32.83 (7.08)	0.28 [−1.05–0.50]	10/10	34.78 (11.51)	33.00 (12.78)	0.15 [−1.02–0.73]	For TG, anxiety was improved significantly, with changes on the DASS-21 anxiety subscale scores (*p* = .02). But no significant changes in STAI state anxiety scores (*p* = .52). For CG, no significantly changes in STAI state and DASS-21 anxiety subscales (*p* = .44; *p* = .15 respectively).
		DASS21	8.62 (7.72)	4.62 (3.95)	0.65 [−1.44–0.14]	7.00 (8.34)	4.80 (5.90)	0.31 [−1.19–0.58]		
Narimani and Musavi (2015)	15/15	DASS21	N/R	N/R	N/C N/C	15/15	N/R	N/R	N/C N/C	MBCT was significantly effective on anxiety of pregnancy women who aged below 20 years (*p* < .0005).
		STAI	N/R	N/R	N/C N/C		N/R	N/R	N/C N/C	
Non-randomized controlled trials										
Dunn et al. (2012)	4/4	DASS2	N/R	N/R	N/C N/C	5/5	N/R	N/R	N/C N/C	1 participant out of 4 participants showed clinical reliable improvement in anxiety in the intervention group after treatment. No participant showed reduction in CG.
Treatment evaluations										
Beddoe et al. (2010)	16/16	STAI-T	36.3 (13.6)	N/R	N/C N/C					Significant reductions in trait anxiety (*p* = .03) from baseline to post intervention, but this reduction was due to lower scores for third-trimester women compared with second trimester women (*p* = .02)
		STAI-S	28.8 (9.7)	N/R	N/C N/C					
Duncan and Bardacke (2010)	27/27	PAS-revised	2.49 (0.58)	2.09 (0.41)	0.80 [−1.35–0.24]					Significant reductions in pregnancy anxiety from pre- to post-intervention (*p* < .0001)
Woolhouse et al. (2014), sample 2	11/11	STAI-S	49.67 (15.22)	39.33 (8.26)	0.84 [−1.72–0.03]					Significant improvements on STAI state scale (*p* = .04), but no significant improvements on DASS anxiety scale (*p* = .20)
		DASS 21	10.20 (2.52)	7.20 (4.54)	0.82 [−1.69–0.05]					
Goodman et al. (2014)	23/23	BAI	12.13 (8.56)	6.35 (4.95)	0.83 [−1.43 - -0.23]					Significant improvements on the BAI scale (*p* < .001)
Byrne et al. (2014)	12/12	DASS21	8.33 (7.57)	6.00 (7.53)	0.31 [−1.11–0.50]					No significant improvements on the DASS21 anxiety subscale after intervention (*p* = .605)
Results for stress symptoms										
RCTs										
Vieten and Astin (2008)	13/13	PSS	20.1 (5.1)	15.9 (5.7)	0.78 [1.57–0.02]	18/18	17.1 (5.0)	16.9 (4.6)	0.04 [−0.70–0.61]	No significant improvement of perceived stress found in the intervention group, compared to the control group (*p* = .35)
Perez-Blasco et al. (2013)	13/13	DASS-21S	18.31 (4.31)	9.54 (6.44)	1.60 (2.31–0.89)	13/8	17.75 (7.44)	18.00 (8.14)	−0.03 (−0.94–0.88)	Large within-subjects reduction in stress scores for Tx; significant decrease in stress in Tx group compared to controls
Guardino (2014)	24/21	PSS	41.81 6.00	37.30 5.38	0.79 [−1.40–0.18]	23/20	39.91 8.55	35.80 8.01	0.50 [−1.10–0.11]	Significant decreases in perceived stress assessed by PSS scale for both groups. No significant difference between intervention group and control group
Woolhouse et al. (2014), sample 1	13/13	PSS	17.92 7.14	16.54 6.12	0.21 [−0.98–0.56]	10/10	16.90 7.08	14.40 8.41	0.32 [−1.20–0.56]	No significant changes on scores of PSS stress scale and DASS21 stress subscale scores in the intervention group from pre- to post-intervention (*p* = .60; *p* = .33, respectively), and in control group (*p* = .18; *p* = .20 respectively)
		DASS21	16.15 11.27	12.92 5.01	0.37 [−1.15–0.41]		13.40 10.79	9.00 4.92	0.53 [−1.42–0.37]	
Narimani and Musavi (2015)	15/15	DASS21	N/R	N/R	N/C N/C	15/15	N/R	N/R	N/C N/C	The results of MANOVA revealed that MBCT is significantly effective on stress of pregnancy women who aged below 20 years (*p* < .0005)
Zhang and Emory (2015)	34/16	PSS	43.9 (10.2)	39.7 (7.46)	0.44 (−0.29–0.59)	31/17	39.5 (8.22)	38.9 (8.62)	0.07 (−0.44–0.58	No difference between Tx and TAU at post-intervention; or at 1 month follow-up
Non-randomized controlled trials										
Dunn et al. (2012)	10/10	DASS	N/R	N/R	N/C N/C	9/9	N/R	N/R	N/C N/C	3/4 of participants in the intervention group experienced clinically reliable decreases in stress symptoms from baseline to post-interventions. In contrast, no participants showed change in DASS stress scale in control group.
Treatment evaluations										
Beddoe et al. (2010)	16	PSS	18.1 4.6	N/R	N/C					Significant decreases in perceived stress from baseline to post intervention (*p* = .05). However, this decline appeared to be from intervention effects on the third trimester group.
Duncan et al. (2010)	27	PSS	26.41 6.73	24.11 4.99	0.40 [−0.93–0.15]					The perceived stress was measured by PSS trended toward improvement after the intervention, but results were not statistically significant (*p* = .062)
Byrne et al. (2014)	12/12	DASS21	9.83 (5.42)	11.50 (6.45)	-0.28 [−0.52–1.08]					No significant improvement of stress measured by DASS-21 after intervention (*p* = .255)
Woolhouse et al. (2014), sample 2	11/11	PSS	22.46 5.79	17.18 5.84	0.91 [−1.79–0.03]					No significant improvements were noted on the PSS (*p* = .09) and DASS stress scale, but show tends toward being significant (*p* = .07).
		DASS21	21.20 9.00	16.60 7.24	0.56 [−1.42–0.29]					

Notes: *S.D.* standard division, *ES* effect size, *95% CI* 95% confidence interval, *Sig.* significant, *N/R* not reported, *N/C* not calculated, *Tx* treatment, *TG* treatment group, *CG* control group, *DASS-21D* Depression, Anxiety and Stress Scale-Depression Subscale, *DASS-21A* Depression, Anxiety and Stress Scale-Anxiety Subscale, *DASS-21S* Depression, Anxiety and Stress Scale-Stress Subscale

In eight non-controlled studies, significant improvements were reported for depressive symptoms after completing MBIs (Battle et al. 2015; Dimidjian et al. 2015; Duncan and Bardacke 2010; Goodman et al. 2014; Muzik et al. 2012; Woolhouse et al. 2014, sample 2). Two non-controlled studies did not find significant reductions in depressive symptoms (Byrne et al. 2014; Felder et al. 2016). Most studies reported moderate to large ESs (Byrne et al. 2014; Dimidjian et al. 2015; Goodman et al. 2014; Muzik et al. 2012; Woolhouse et al. 2014, sample 2), while one study showed a small ES (Duncan and Bardacke 2010) and one showed a negligible effect (Byrne et al. 2014, *d* = 0.08).

With regard to anxiety, 12 studies examined the effectiveness of MBIs on anxiety symptoms (Table 3). Included RCTs (*n* = 7) suggested that participants engaging with MBIs showed significant reductions in anxiety compared with controls (all *p* < .05, Guardino et al. 2014; Narimani and Musavi 2015; Perez-Blasco et al. 2013; Vieten and Astin 2008; Woolhouse et al. 2014, sample 1). One non-randomized controlled study reported that one out of four participants was free of anxiety symptoms after treatment compared to none in the control group (Dunn et al. 2012). Of the five controlled studies, three RCTs reported data convertible to ESs. Most of the effects were of moderate to large size (Guardino et al. 2014; Perez-Blasco et al. 2013; Vieten and Astin 2008; Woolhouse et al. 2014, sample 1). In the one study that assessed pregnancy-related anxiety, there was a small effect size (Guardino et al. 2014). Four out of five non-controlled studies suggested significant improvements of anxiety after treatment (Beddoe et al. 2010; Duncan and Bardacke 2010; Goodman et al. 2014; Woolhouse et al. 2014, sample 2). However, two studies reported reductions in anxiety that did not reach statistical significance (Byrne et al. 2014; Woolhouse et al. 2014, sample 2), although in one study, a large ES was observed (Woolhouse et al. 2014, sample 2). These studies both used the DASS-21 to measure anxiety. Three studies showed large ESs, while one showed a small to moderate ES (*d* = 0.31) (Byrne et al. 2014).

**Table. 3:** Assessment of risk of bias

Study (authors, years	Selection bias			Performance bias		Detection bias		Attrition bias		Reporting bias	Total (max. 10)
	Adequate random sequence generation N/A if not RCT	Adequate allocation concealment N/A if not RCT	Similar baseline characteristics N/A if not RCT	Adequate participants blinding	Adequate treatment provider blinding	Adequate outcome assessor blinding	Similar timing of outcome assessment	Acceptable and described drop-out rate	Inclusion of an intervention-to treat analysis	No selective outcome reporting	
Vieten and Astin (2008)	Yes	Unclear	Yes	Unclear	Unclear	Unclear	Yes	Yes	No	Yes	5
Beddoe et al. (2010)	N/A	N/A	N/A	Unclear	Unclear	Unclear	Yes	Yes	No	Yes	3
Duncan and Bardacke (2010)	N/A	N/A	N/A	Unclear	Unclear	Unclear	Yes	Yes	No	Yes	3
Dunn et al. (2012)	Unclear	Unclear	Yes	Unclear	Unclear	Unclear	Yes	Unclear	No	Yes	3
Muzik et al. (2012)	N/A	N/A	N/A	Unclear	Unclear	Unclear	Yes	Yes	No	Yes	3
Perez-Blasco et al. (2013)	Unclear	Unclear	Yes	Unclear	Unclear	Unclear	Yes	Yes	No	Yes	4
Guardino et al. (2014)	Yes	Unclear	Yes	Unclear	Unclear	Unclear	Yes	Yes	No	Yes	5
Woolhouse et al. (2014), study 1	Yes	Yes	Yes	No	Unclear	Unclear	Yes	Yes	No	Yes	6
Woolhouse et al. (2014), study 2	N/A	N/A	N/A	No	Unclear	Unclear	Yes	Yes	No	Yes	3
Goodman et al. (2014)	N/A	N/A	N/A	Unclear	Unclear	Yes	Yes	Yes	No	Yes	4
Byrne et al. (2014)	N/A	N/A	N/A	Unclear	Unclear	Unclear	Yes	Yes	No	Yes	3
Battle et al. (2015)	N/A	N/A	N/A	Unclear	Unclear	Unclear	Yes	Yes	No	Yes	3
Dimidjian et al. (2015)	N/A	N/A	N/A	Unclear	Yes	Yes	Yes	Yes	Yes	Yes	6
Narimani and Musavi (2015)	Yes	Unclear	Yes	Unclear	Unclear	Unclear	Yes	Unclear	No	Yes	4
Miklowitz et al. (2015)	N/A	N/A	N/A	No	No	No	Yes	Yes	Yes	Yes	4
Zhang and Emory (2015)	Unclear	Unclear	Unclear	Unclear	Unclear	Unclear	Yes	No	Yes	Yes	3
Dimidjian et al. (2016)	Unclear	Unclear	Yes	Unclear	Unclear	Yes	Yes	Yes	Yes	Yes	6
Felder et al. (2016)	N/A	N/A	N/A	No	No	No	No	Yes	Yes	Yes	3

*Note*: the appendix shows the results for methodological quality assessment for each included study and reporting risk of bias for each included study. *N/A* not applicable, the answer ‘yes’ coded = 1, ‘unclear’ and ‘no’ = 0 score. The total score is 10. Higher scores indicate lower risk of bias

Of the included studies, six RCTs, one non-randomized controlled study and four non-controlled studies assessed pre- to post-treatment changes in stress (Table 2). Within-subject ESs suggested large pre-post ESs for reduction in stress (three studies) (Guardiano et al. 2014; Perez Blasco et al. 2013; Vieten and Astin 2008), and two studies suggested reductions of moderate magnitude (Woolhouse et al. 2014, sample 1, DASS-21; Zhang and Emory 2015). However, one study reported a small effect using the Perceived Support Scale (PSS; Woolhouse et al. 2014, sample 1). One study reported data that could not be converted to give ESs (Narimani and Musavi 2015). Results were more equivocal when MBIs were measured against a control. Here, only one study suggested a significant effect favoring MBI (Perez-Blasco et al. 2013). In addition, the one non-randomized controlled study (Dunn et al. 2012) reported 75% of the treatment group reported a clinically reliable reduction in stress, while none of the control participants showed reductions in stress. In terms of outcomes from non-controlled studies, the results are similarly equivocal. One study (Beddoe et al. 2010) reported a significant decrease in perceived stress over time (*p* = .05), which they proposed was related to the third trimester group. One study (Woolhouse et al. 2014, sample 2) showed post-treatment improvements on the PSS and DASS21 stress scale at trend level (*p* = .09 and *p* = .07) but with moderate to large ESs. In addition, Duncan and Bardacke (2010)reported post-treatment PSS reductions of small to moderate ES but not reaching statistical significance (*p* = .062). Finally, Byrne et al. (2014) reported DASS21 stress scores increased over time, consistent with a small negative ES.

Thirteen studies assessed changes in mindfulness. Five RCTs provided evidence of greater mindfulness after treatment compared to controls, consistent with medium to large ESs (Felder et al. 2016; Guardino et al. 2014; Perez Blasco et al. 2013; Woolhouse et al. 2014, sample 1; Zhang and Emory 2015). The one non-randomized controlled study (Dunn et al. 2012) reported that one participant out of four participants showed clinical reliable improvement in MASS mindfulness in treatment group versus no participant in the control group. For non-controlled studies, five out of six non-controlled studies showed pre-post treatment increases in mindfulness scores on at least one subscale of the Five Facet Mindfulness Questionnaire (FFMQ; Baer et al. 2006) after treatment (*p* < .05 for all; Battle et al. 2015; Duncan and Bardacke 2010; Goodman et al. 2014; Muzik et al. 2012; Woolhouse et al. 2014, sample 2). However, the magnitude of ES varied, with small ESs in two studies (Goodman et al. 2014; Muzik et al. 2012) and moderate to large ESs in the remaining studies (Battle et al. 2015; Duncan and Bardacke 2010; Woolhouse et al. 2014, sample 2). The majority of studies that used the FFMQ reported total scores consistent with moderate to large ESs (Duncan and Bardacke 2010; Muzik et al. 2012; Perez-Blasco et al. 2013; Vieten and Astin 2008; Woolhouse et al. 2014, sample 2).

Six studies provided quantitative data for the long-term effects of MBIs at follow-ups of up to 6 months. Dunn et al. (2012) reported that approximately half of the treatment group participants showed improvement in stress and half the participants showed improvement in depression assessed by EPDS, while these changes were not observed in the control group. In Zhang and Emory’s (2015) study, a greater decrease in depressive symptoms was noted in the MBI group at 1-month follow-up, compared to controls. Furthermore, two studies with clinical samples (Dimidjian et al. 2016; Miklowitz et al. 2015) reported lower levels of depressive symptoms in MBCT participant compared to controls at 6-month follow-up. However, Vieten and Astin (2008) reported no significant improvements in depression and anxiety between treatment group and control group, while Guardino et al. (2014) did not find sustained treatment effects at the 6-week follow-up. Three studies reported findings with regard to relapse of depressive symptoms (Dimidjian et al. 2015, 2016; Miklowitz et al. 2015). All three studies reported that MBCT was effective in reducing depressive relapse rates post-intervention with recurrence rates of between 18 and 22%. These outcomes were maintained at up to 6 months postpartum (Dimidjian et al. 2016).

Of the included studies, 16 studies reported the dropout rates. In the RCTs, dropout rates in the treatment group were relatively lower than in the control group, with the exception of one socio-economic high-risk sample (Zhang and Emory 2015) and an online web program trial (Felder et al. 2016). From the non-controlled studies, dropout was also relatively low with a range from 4% (Goodman et al. 2014) to 45% (Woolhouse et al. 2014, sample 2).

Finally, the results of the risk of bias evaluation are presented in Table 3. Of the 17 included studies, there was considerable variability in the spread of risk of bias ratings, with RCTs reporting greater adherence to attempts to minimize bias. However, there was a spread of ratings with some RCTs having risk of bias ratings similar to non-controlled studies. With regard to methodological aspects, there was evidence that selection bias, performance bias and assessor blinding were generally more consistently omitted or unclear in the included studies. In contrast, all studies reported similar timing of outcome assessments. Most of studies reported dropout rates, and intention-to-treat analyses were used in most of the more recent RCTs (Dimidjian et al. 2015, 2016; Miklowitz et al. 2015). Risk of reporting bias was low in all studies.

## Discussion

Our review systematically reviewed the evidence for the effectiveness of MBIs on perinatal depression and anxiety. Outcomes for depression and stress show some evidence of treatment effects, although this was less pronounced in studies comparing MBIs to control groups. The treatment effects of MBIs on anxiety were more consistent and of greater magnitude than the effects of MBIs on depression and stress and were observed across differing study designs. Most studies reported increased mindfulness post treatment, suggesting face validity of the intervention. Although only measured in a minority of studies, there was a small evidence base for the long-term effects of MBIs, particularly in relation to recurrence of depression. This may therefore be a promising avenue for future studies in the area. Taken as a whole, the evidence base suggests that MBIs have high acceptability, as measured by attendance in both general population samples and mental health samples. In the RCT and non-RCT studies, the dropout rates for MBIs appeared lower compared with control groups.

Our findings for anxiety are consistent with previous evidence that MBIs are effective in reducing symptoms of anxiety disorder (e.g. Hofmann et al. 2010; Hoge et al. 2013; Koszycki et al. 2007). It may be the case that mindfulness practice decreases cognitive aspects of anxiety via decreased frequency of negative automatic thoughts (Frewen et al. 2008) or via the impact on physiological arousal. For instance, preliminary evidence suggests that MBIs promote sleep quality for pregnant women (Beddoe et al. 2010). As anxious arousal in the perinatal period may be linked to over-activity of the HPA in infants (Talge et al. 2007), it is also possible that the decreased anxiety associated with MBI may benefit the infant via reduced maternal distress and better regulation of HPA arousal (Salmon et al. 2009).

In contrast to the findings in adult non-pregnant samples (e.g. Hoffman et al. 2010), the review did not find clear associations between MBI and reductions in depressive symptoms. There are several possible explanations for this inconsistent pattern of findings. Under-powering due to small sample size was an issue with several studies showing large but non-significant ESs (e.g. Vieten and Astin 2008). In addition, MBCT was originally designed as an intervention for recurrent depression (Segal 2002). However, most of the samples in the current review had lower levels of baseline depression severity. Therefore, the failure to detect significant change may represent a floor effect. In addition, all reviewed studies that found non-significant treatment effects on depression involved general population samples, with below cut-off scores on depression measures. However, results for non-controlled studies from perinatal mental health samples showed significant remission of depression after treatment (e.g. Woolhouse et al. 2014). Studies also relied on a diverse range of self-report measures, thus increasing heterogeneity. It is also the case that symptom measures used in the studies may be related to the non-significant outcomes for depression. There also remains the possibility that, despite the sensitivity and specificity of self-report measures for identifying depression (e.g. Thomas et al. 2001), general measures such as the CES-D may not be adequate to identify depression and anxiety in pregnant or postnatal women, due to the overlap between somatic symptoms of pregnancy and certain items of depression measures (e.g. lack of energy). One alternative would be to use of pregnancy-specific measures of low mood (e.g. EPDS) in conjunction with general measures of depression. We also note that the demographic characteristics of the majority of samples suggested a bias towards relatively well-educated women in stable relationships. This applied to both general population and mental health samples. Therefore, adaptations to the delivery of MBIs may be required to target low-income families or women experiencing multiple adversities.

We acknowledge that the review was limited by the number of studies available and variability in the methodological quality of the primary studies. This heterogeneity led us to focus on a narrative synthesis, rather than conduct a meta-analysis of the results. The included studies varied widely on validation methods, study design, data reporting, severity of mental health difficulties and gestation weeks at baseline, therefore restricting comparisons between studies. We also note that limiting inclusion to studies published in English may have led to the omission of papers. However, Taylor et al. (2016) have recently meta-analyzed the mindfulness studies included in this review. Given the rapid growth of literature in this area, it would be reasonable to conduct a further analysis as the literature increases. We also acknowledge that our review combines samples recruited due to their mental health status and general maternity samples. This introduces methodological variance into the synthesis of the results. However, we contend that this ambiguity reflects different care pathways with regard to the assessment and monitoring of mental health in pregnancy, which would be lost with a more stringent focus on inclusion criteria. We also note ambiguity in the primary studies regarding the measurement of depression which was largely based on self-reported depressive symptoms—although the EPDS was used in the majority of studies. Therefore, further research using interview-validated diagnostic measures of low mood would be merited to increase the rigor of assessment of mental health in this area. Furthermore, the review is limited by the lack of follow-up studies to test the long-term effects of MBIs and qualitative results. Finally, we also observe that study risk of bias was variable, but this was not a simple case of all RCTs having reduced bias compared to non-controlled and treatment evaluation studies. Most studies used appropriate analyses, but improvements could be made to the reporting of randomization, blinding and controlling for dropout. We suggest that these difficulties are common across many health service-based treatment evaluations in perinatal and infant mental health (e.g. MacBeth et al. 2015).

In terms of future research and practice, our review highlights the need for more methodologically rigorous trials of MBIs in the perinatal period. This includes greater clarity around the optimal target population for intervention. Much of the research we review used general population samples, suggesting a role for a generalized MBI for wellbeing in pregnancy. However, from a mental health perspective, it may be more effective to target interventions at women meeting ‘high-risk’ criteria for mental health in pregnancy due to current or previous psychiatric symptoms. Furthermore, trials would benefit from incorporating interview-based or diagnostic measures of mental disorder into trial protocols. In addition, the role of MBIs in preventing depressive relapse, which was a key driver in the development of MBCT for depression, remains under evaluated. A further consideration with regard to targeting of interventions lies within the differing motivations for engagement with interventions between women from the general population without symptoms of distress compared to women presenting with past or current mental health difficulties. Indeed, the literature on common mental health difficulties in pregnancy highlights that this is likely to be the case (Staneva et al. 2015b). As such, there is scope for qualitative assessment of women’s motivations and experiences of MBIs in pregnancy. We also note that the majority of studies were conducted in the USA or Australia. Given the increasing ubiquity of mindfulness practice, it would be beneficial for future studies to be conducted in other settings. Further research is also required with regard to long-term effects of MBIs on maternal and child outcomes. Limitations notwithstanding, our review suggests that MBIs are a non-pharmacological approach to maternal distress likely to be acceptable to women in pregnancy and could therefore be integrated into existing programs of pregnancy care for both with additional targeted adaptations for ‘high-risk’ groups.
